# Cholecystokinin and Somatostatin Negatively Affect Growth of the Somatostatin-RIN-14B Cells

**DOI:** 10.1155/2009/875167

**Published:** 2008-12-01

**Authors:** Karim El-Kouhen, Jean Morisset

**Affiliations:** Service de Gastroentréologie, Département de Médecine, Faculté de Médecine, Université de Sherbrooke, Sherbrooke, QC, Canada J1H 5N4

## Abstract

With the exclusive presence of the pancreatic CCK-2 receptors on the pancreatic delta cells of six different species, this study was undertaken to determine the role of cholecystokinin and gastrin on growth of these somatostatin (SS) cells. For this study, the SS-RIN-14B cells were used in culture and their growth was evaluated by cell counting. *Results*. To our surprise, we established by Western blot that these RIN cells possess the two CCK receptor subtypes, CCK-1 and CCK-2. Occupation of the CCK-1 receptors by caerulein, a CCK analog, led to inhibition of cell proliferation, an effect prevented by a specific CCK-1 receptor antagonist. Occupation of the CCK-2 receptors by the gastrin agonist pentagastrin had no effect on cell growth. Proliferation was not affected by SS released from these cells but was inhibited by exogenous SS. *Conclusions*. Growth of the SS-RIN-14B cells can be negatively affected by occupation of their CCK-1 receptors and by exogenous somatostatin.

## 1. Introduction

In the pancreas, the cholecystokonin-2 receptor (CCK-2R), formerly the CCK-B receptor, has been located exclusively on the somatostatin-delta cells of six different species, including man [1]. To date, nothing is known about the role of this CCK receptor subtype on the physiology of these delta cells. It has been reported however that chronic injections of caerulein, a CCK analog, and secretin caused significant increases in rat pancreatic somatostatin (SS) content without any effect on total delta cell numbers [2]. Similar changes in SS contents were also observed with the same hormones combination in islets cultured for 10 days with no difference in islets' total DNA contents [3].

It was initially demonstrated that gastrin and CCK-8, at relatively high doses, caused a quick and transient twofold increase in SS release by the perfused canine pancreas [4]. When CCK-8 was infused at a dose that stimulates the secretion of protein in the pancreatic juice of dogs to the levels normally observed with a meal [5], SS in the pancreatic vein rose immediately about 2-fold above basal and remained elevated for the 15 minutes of the hormone infusion [6]. These data indicate that gastrin and CCK can induce SS secretion from the pancreatic gland which was later directly confirmed from rat islet cell in culture [7].

In order to have an exclusive and direct access to the pancreatic somatostatin cells and to avoid mixtures of cell types from islet cell cultures, investigators established and cloned a transplantable rat islet cell tumor which secretes insulin and somatostatin [8]. Clones of the original somatostatin cell line were used to study somatostatin secretion (RINT3), intracellular signalling pathways activated in response to CCK receptor subtypes occupation (RIN1027-B2) and CCK receptor subtypes characterization and SS release (RIN-14B) [9–11].

This study was undertaken to establish the presence of the two CCK receptor subtypes on the RIN-14B cells and to determine if one or both of these types can affect growth of these cells. To our knowledge, no study has yet been performed to explore if the gastrointestinal hormones CCK and gastrin, two specific CCK-1 and CCK-2 receptors agonists, respectively, have any effects on growth of the pancreatic somatostatin cells.

## 2. Materials and Methods

**Products**

Somatostatin-14 (SS-14) was purchased from Bachem Bioscience (King of Prussia, Pa, USA). Pentagastrin (PG) was purchased from Sigma-Aldrich (Oakville, ON, Canada). Caerulein (Cae) was generously given by Dr. R. de Castiglione, Farmitalia (Milan, Italy). L-364,718 and L-365,260 were gifts from Dr. V. J. Lotti, Merck Sharp and Dohme Research Laboratories (West Point, Pa). The RIN-14B cells were obtained from ATCC (Manassas, Va, USA). The COS cells were gifts from Dr. M. Tremblay (McGill Cancer Centre, Montreal, Canada). Cell culture media reagents were obtained from Life Technologies (Burlington, ON, Canada). The somatostatin (Barbar) antibody, purified by affinity chromatography, was a gift from Dr. P. Brazeau, Université de Montréal, Montreal, Canada. The goat polyclonal anti-CCK-1 receptor antibody (7509) was raised against the rat internal peptide (KFDASQKKSAKEKR) and the goat polyclonal anti-CCK-2 receptor antibody (6767) was raised against the human internal peptides CCK-2 (FDGDSDSDSQSRVRNQ). These two antibodies and corresponding peptides were gifts from Dr. J. Voskuil (Everest Biotech, Oxfordshire, UK).

**Cell Culture**

The rat RIN-14B cell line is a secondary clone derived from the RIN-m rat islet cell line; these cells synthesize and secrete somatostatin [8]. Routinely, the cells were grown on Petri dishes in RPMI 1640 (for RIN-14B) and DMEM (for COS cells) media containing 25 mM glucose, 10% fetal bovine serum (FBS), penicillin (100 UmL^−1^), streptomycin (100 *μ*g mL^−1^), 2 mM L-glutamine, 10 mM HEPES, 1 mM sodium pyruvate, and 1.5 g L^−1^ sodium bicarbonate. Cells were kept in culture at 37°C in a 5% CO_2_ humidified atmosphere. The culture media were changed every other day; they were passed weekly by trypsin (0.25%)-EDTA (2.2 mM) detachment and subcultured every week at a ratio of 1 : 7.

**Growth Assay**

Cells were plated in 60 mm diameter Petri dishes at a density of 0.2 × 10^6^ mL^−1^ (4 mL/dish). The next day, cells were transferred to RPMI 1640-0.5% heat inactivated FBS and adapted overnight to grow in this medium before addition of peptides, antiserum, and CCK-receptor antagonist. The medium was changed daily and cells were supplemented also daily with Cae (10^−10^ M), PG (10^−5^ M), and SS-14 (10^−9^ M) alone or in combination with L-364,718 (10^−7^ M) or Barbar (0.25 *μ*g mL^−1^). Cell growth was measured after 1, 2, 3, 4, and 5 days with a cell counter (Coulter counter).

**SDS-PAGE and Western Blot**

The membranes of the RIN-14B and COS cells were prepared as described in [12]. Briefly, cells were resuspended in a homogenization buffer (10 mM HEPES, pH 7.5, 250 mM sucrose, 1 mM EGTA, 1 mM EDTA, 0,5 mM PMSF, 20 *μ*M leupeptin, 1 *μ*M aprotinin, and 2 *μ*M pepstatin), homogenized using a Potter homogenizer and sonicated for 10 seconds (40% power). The membranes were then collected by centrifugation at 30 000 g for 30 minutes at 4°C using a Beckman TLS-55 rotor (Palo Alto, Calif, USA) and resuspended at 17 mg mL^−1^ in the homogenization buffer. Proteins were determined with the BCA assay of Pierce using bovine serum albumin as standard.

Membrane proteins (100 *μ*g) were separated by electrophoresis on a 10% SDS-polyacrylamide gel according to [13] and transferred to nitrocellulose membranes (Sigma-Aldrich). The membranes were blocked for 2 hours with NAP-blocker (G Biosciences, St-Louis, Mo, USA) in TBST (1 : 2 v/v) and incubated overnight at 4°C in NAP-blocker: TBST (1 : 2 v/v) with either the anti-CCK-1R 7509 (0.1 *μ*g mL^−1^) or the anti-CCK-2R 6767 (0.1 *μ*g mL^−1^) antibodies. The blots were then washed in TBST and incubated for 1 hour at RT° in NAP-blocker: TBST (1 : 2 v/v) with horseradish peroxidase-conjugated antigoat (1 : 10 000). Blots were washed with TBST and proteins were detected by chemiluminescence using ECL immunodetection system (Amersham Biosciences, Baie d’Urfé, QC). Specificity of each CCK receptor antibody was established by preincubation of each antibody for 2 hours at RT° with its specific peptide antigen: 1 *μ*g mL^−1^ of 7509 and 0.1 *μ*g mL^−1^ of 6767 peptides.

**Statistical Analysis**

Results were analyzed by the Student's *t*-test for comparison of independent samples with a probability value of <.05 which is considered significant.

## 3. Results

### 3.1. Presence of the CCK Receptor Subtypes on the RIN-14B Cells

**Figure 1:**
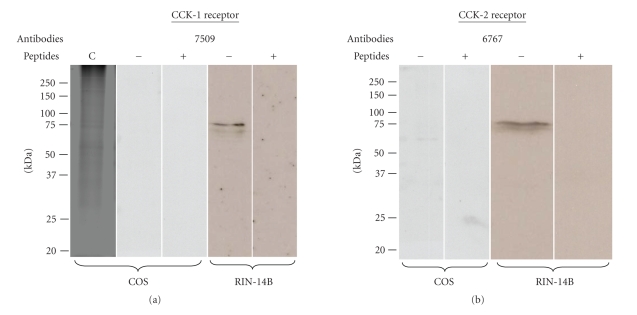
*Specificity of the CCK-1 and CCK-2 receptor antibodies established by Western blotting.* Membrane from the RIN-14B and COS cells (100 *μ*g) were used for Western blotting analysis with antibodies 7509 (0.1 *μ*g mL^−1^) and 6767 (0.1 *μ*g mL^−1^). Specificity was established by preincubation of each primary antibody for 2 hours at RT with each receptor-specific peptide antigen (1 *μ*g mL^−1^ of 7509 and 0.1 *μ*g mL^−1^ of 6767 peptides). C: blue Coomassie gel coloration.

To establish the presence of both CCK receptor subtypes on the RIN-14B cells, we used specific antibodies raised against both receptors. With the specific CCK-1 receptor antibody (7509), we can see that the RIN-14B cells express the CCK-1 receptor as a 75 kDa protein. This recognition by the antibody is specific because the band totally disappeared with preincubation of the antibody with the peptide used to develop this antibody (see Figure 1(a)). Similarly, these cells also express the CCK-2 receptor, also as a 75 kDa protein identified with the antibody 6767; specificity of the receptor-antibody binding was also revealed with preincubation of the antibody with the peptide with disappearance of the band (see Figure 1(b)).

To further strengthen the specificity of both CCK-1 and CCK-2 receptor antibodies, we established the absence of these two CCK receptors in COS cells known to be free of these two receptors [14]. As shown in Figures 1(a) and 1(b), both antibodies 7509 (anti-CCK-1) and 6767 (anti-CCK-2) did not recognize the 75 kDa band observed in the RIN-14B cells. This observation further supports the specificity of the two CCK receptor antibodies we used in this study to demonstrate their presence in the RIN cells.

### 3.2. Effect of Caerulein on RIN-14B Cell Growth

**Figure 2:**
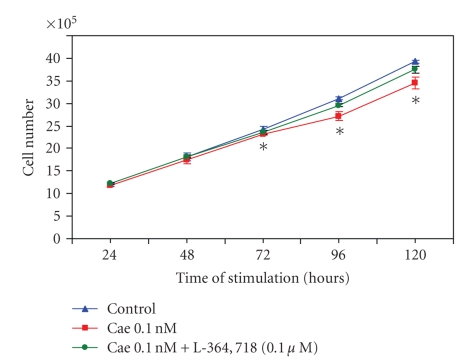
*Growth control of the RIN-14B cells by the CCK-1 receptor agonist caerulein (Cae) and the receptor's specific antagonist, L-364718.* Cells were grown for up to 120 hours in the presence of 0.1 nM caerulein ± 0.1 *μ*M L-364718. Medium and drugs were changed daily and cells were counted daily for up to 5 days. In this experiment, results represent data collected from two separate experiments with four wells per group; overall they represent 8 different wells in each group. *Significantly different than control at *P* < .05.

To determine if the CCK-1 analog caerulein can affect the growth of the RIN-14B cells, growth assays were performed. As shown in Figure 2, caerulein, used at a concentration which totally occupies the high affinity CCK-1 receptors and causing maximal amylase release from rat dispersed pancreatic acini [15], caused significant reductions in RIN-14B cell growth from day 3 to day 5 of culture. The percentage of growth inhibition at day 5 reached 12%. The observation that this inhibition by caerulein was totally reversed by the specific CCK-1 receptor antagonist L-364718 indicates that the CCK-1 receptors are involved in this process of growth inhibition. To eliminate the possibility that somatostatin released in response to caerulein [11] could be responsible for growth inhibition by the CCK analog, cells were also incubated with an SS antibody along with caerulein; in no time did we observe a difference between growth in the caerulein group alone and caerulein plus the SS antibody (data not shown). As shown in Figure 4, the SS antibody prevented growth inhibition by SS alone.

### 3.3. Effect of Pentagastrin on RIN-14B Cell Growth

**Figure 3:**
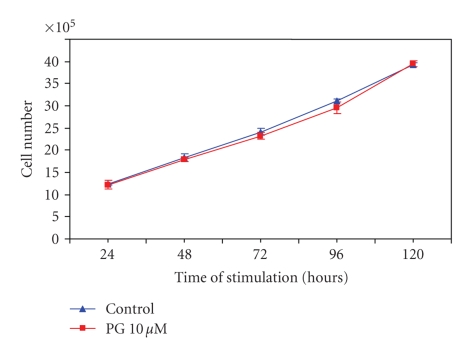
*Growth control of the RIN-14B cells by the CCK-2 receptor agonist pentagastrin (PG).* Cells were grown for up to 120 hours in the presence of 10 *μ*M PG. Medium and PG were changed daily and cells were counted daily for up to 5 days. Results are the means ± SE of 5 wells per point in each group.

To establish if the CCK-2 agonist pentagastrin could affect growth of these RIN-14B cells, growth assays were also performed. The results indicate that contrary to what was observed with occupation of the CCK-1 receptors, occupation of the CCK-2 receptors by 10 *μ*M pentagastrin, a gastrin analog, had no effect on RIN-14B cell growth over the 5-day period examined (see Figure 3). This strongly indicates that occupation of the CCK-2 receptors did not alter the growth of the RIN-14B cells.

### 3.4. Effect of Somatostatin on RIN-14B Cell Growth

**Figure 4:**
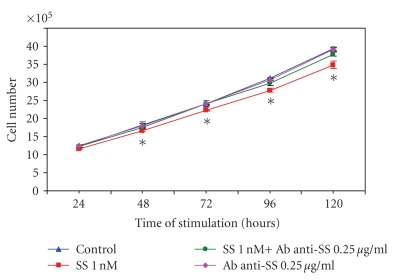
*Growth control of the RIN-14B cells by somatostatin and a specific somatostatin antibody.* Cells were grown for up to 120 hours in the presence of 1 nM somatostatin (SS) ± 0.25 *μ*g mL^−1^ of SS antibody (Ab anti-SS) or Ab anti-SS alone. Medium and drugs were changed daily and cells were counted daily for up to 5 days. Results are the means ± SE of 5 wells per point in each group. *Significantly different than control at *P* < .05.

To verify if somatostatin could have an inhibitory effect on growth of the cells responsible for its synthesis, the RIN-14B cells were incubated for up to five days in the presence of 1 nM somatostatin-14. As shown in Figure 4, somatostatin significantly reduced growth of the RIN-14B cells by 11.3% after 5 days of culture; this inhibitory effect was already significant after 2 days of culture. Addition of a somatostatin antibody to the culture medium to neutralize endogenous SS released by the cells had no effect on their growth; however its addition to exogenous somatostatin reversed its growth inhibition. These results indicate that endogenous somatostatin released from these cells did not reach the concentration needed to inhibit their growth but when applied at the needed concentration, exogenous somatostatin can inhibit their growth.

## 4. Discussion

This study reports for the first time that (1) the SS-secreting RIN-14B cells express the two known and characterized CCK receptor subtypes; (2) occupation of the CCK-1 receptor subtype leads to inhibition of these cells' growth, while occupation of the CCK-2 receptor subtype fails to stimulate or inhibit such growth; (3) somatostatin can also inhibit growth of these RIN-14B cells probably through an autocrine mechanism.

In contrast to substances which can control somatostatin secretion, nothing is currently known about those responsible for delta cells' growth. The observation that the RIN-14B cells express the two CCK receptor subtypes is not unique to these cells. Indeed, expression of both receptor subtypes has been documented in normal rat pancreatic acini with a predominance of the CCK-1 subtype over that of the CCK-2 [16]. These two receptors were also reported in normal human and rat islets but on different cell types, the CCK-1 receptors on alpha and beta cells and the CCK-2 receptors on the delta cells using two different antibodies [17]. Studies performed in rats demonstrated that in pancreatic malignancies, the CCK-1 receptor is overexpressed while the CCK-2 receptor is newly expressed [18]. In human pancreatic tumors however, the distribution of the two CCK receptor subtypes is still controversial. Indeed, by using the PCR technique, one study reported the presence of the CCK-2 receptors in all samples of normal pancreas and pancreatic adenocarcinoma; the CCK-1 receptor expression could not be detected in normal pancreatic samples but it appeared in all samples of pancreatic adenocarcinomas [19]. By receptor autoradiography, the CCK-2 receptor was found occasionally in pancreatic tumors while the CCK-1 receptor was mostly expressed in these tumors [20]. These data emphasize that the expression of these two CCK receptor subtypes in many cancer cell types may be an important indicator of the influence of CCK and gastrin of local or systemic origin on the growth of these cancers.

Indeed, in Elas CCK-2 receptor transgenic mice, the growth rate of their pancreas was increased by 40% after birth between 40 and 110 days of age; this expression had a key role in the development of pre- and neoplastic lesions in their pancreas [21]. While everyone agrees that occupation of the CCK-1 receptors in the pancreas of rats [22] and other rodents led to growth of the organ, its presence and stimulation in MiaPaCa-2 and Panc-1 cells led to growth inhibitory responses [23]. Occupation of the CCK-2 receptor also resulted in surprising opposite effects when transfected in CHO and Swiss 3T3 cells. It inhibited proliferation and DNA synthesis in the CHO-CCK-2 cells while stimulation occurred in the Swiss 3T3-CCK-2 cells; these opposite effects on growth happened while CCK-8 stimulated the same common second messenger pathways [24]. Growth inhibition was also observed with occupation of the transfected CCK-2 receptors in the human pancreatic MiaPaca-2 and Panc-1 cells [23]. Interestingly, coexpression of gastrin and CCK-2 receptors were observed in 5/5 and 7/8 human gastric and colorectal cell lines and these cells maintain an autocrine growth pathway [25]; the RIN-14B cells also express gastrin although in small quantities (data not shown).

When comparing all the above results with those obtained with the RIN-14B cells, it seems that growth inhibition observed in response to caerulein in this study renders these RIN cells comparable to the transfected CCK-1 MiaPaca-2 and Panc-1 cells [23]. Endogenous CCK also resulted in growth inhibition of human cholangiocarcinoma; however, no specific antagonists of either CCK receptor subtypes were used to confirm which CCK receptor was involved [26]. This study points out that CCK can inhibit growth not only in cells transfected with both CCK receptor subtypes. Moreover, the inhibition observed in these RIN cells really resulted from occupation of the CCK-1 receptors because it has been reversed by L-364718, a specific CCK-1 receptor antagonist.

Somatostatin can inhibit endogenous SS secretion from the delta cells through an auto-feedback mechanism [27]. Although we have not investigated which somatostatin receptors are present on the RIN-14B cells, it has been reported that about 70% of the rat pancreatic delta cells express the SS receptor subtypes 1–4 [28].

Since it is accepted that the antiproliferative effect of somatostatin results from its action via the endocrine pathway, evidence also exists that somatostatin can also act via an autocrine/paracrine pathway which has been recently described in PC-3 and LNCaP cells, two human prostate adenocarcinoma cell lines [29]. We do not believe that growth of these RIN-14B cells is autoregulated by somatostatin they release into the medium. This conclusion is based on the following observations: (a) these cells grew in a 0.5% inactivated FBS medium when they release into the medium approximately 200 pg mL^−1^ of somatostatin per 4 hours [11], and (b) they also grew at their control rates even in the presence of 0.25 *μ*g mL^−1^ of a specific somatostatin antibody; an autocrine regulated pathway would have shown these cells grow at a rate above the controls in the presence of the antibody. This was previously observed in vivo when the trophic effect of caerulein on the rat pancreas was significantly enhanced by a simultaneous administration of a somatostatin antiserum, the same used in this study [30].

Although endogenous somatostatin released by the RIN cells does not seem sufficient to sustain growth inhibition, these cells are however sensitive to somatostatin as they exhibited growth inhibition in its presence at a higher concentration. The magnitude of inhibition is comparable to what was previously described in AR4-2J cells where at 10 nM, the hormone caused a 25% growth inhibition over a 96-hour period [31]. This inhibitory effect of somatostatin on the RIN cells proliferation is specific since prevented by the presence of the somatostatin antibody.

In conclusion, our data indicate for the first time that the somatostatin RIN-14B cells possess the two CCK receptor subtypes, and that their proliferation can be negatively affected by occupation of the CCK-1 receptor subtype and exogenous somatostatin.
